# EVALUATING RELAPSE PROPHYLAXIS IN ADDITION TO INTERDISCIPLINARY MULTIMODAL PAIN THERAPY FOR BACK PAIN: A RANDOMISED CONTROLLED TRIAL

**DOI:** 10.2340/jrm.v57.42088

**Published:** 2025-08-20

**Authors:** Julia SCHMETSDORF, Kathrin KRÜGER, Jacqueline POSSELT, Werner RUNDE, Hans-Georg ZECHEL, Thomas KOHLMANN, Christian KRAUTH

**Affiliations:** 1 Hannover Medical School, Institute for Epidemiology, Social Medicine, and Health Systems Research, Hannover; 2 Centre for Health Economics Research Hannover (CHERH), Hannover; 3 AOK Niedersachsen, Hannover; 4 Rehabilitation Centre, Outpatient Department for Orthopedic Rehabilitation, Bad Zwischenahn; 5 University of Greifswald, Institute for Community Medicine, Greifswald, Germany

**Keywords:** back pain, pain management, quality of life, randomized controlled trials as topic, rehabilitation, secondary prevention, sick leave

## Abstract

**Objective:**

This study aimed to evaluate the effectiveness of a 12-month relapse prophylaxis following a 4-week interdisciplinary multimodal pain therapy approach for patients with back pain. The study examined whether the intervention reduced days of incapacity to work (primary outcome) and improved functional capacity and health-related quality of life (secondary outcomes) compared with interdisciplinary multimodal pain therapy alone.

**Design:**

A randomized controlled trial was conducted. The recruitment period was 24 months.

**Subjects/Patients:**

The study comprised 297 employed patients from a rural region in north-west Germany, diagnosed with back pain in different regions of the spine.

**Methods:**

The analyses were based on quantitative data: claims data and questionnaire data.

**Results:**

The results showed a mean of 70.07 days of incapacity to work after the interdisciplinary multimodal pain therapy for the control group and a lower mean of 56.41 days for the intervention group. The group difference was not significant (*p* = 0.259). Analysis of change scores revealed statistically significant larger improvements of functional capacity and health-related quality of life in the intervention group.

**Conclusion:**

Findings of this study show improvements in the secondary outcomes. The results indicate that further studies are needed to determine how to sustainably reduce days off work due to back pain.

## LAY ABSTRACT

Back pain reduces patients’ quality of life and leads to high costs for society. Two groups of patients were compared in this study. Both groups received an interdisciplinary back training programme and 1 group also received follow-up treatment. The results showed that the intervention group had fewer sick days and better functional capacity and quality of life. Although improvements were found, the effectiveness and implementation of an aftercare programme should be further investigated.

A total of 26.2 million people in Germany were diagnosed with back pain in 2021, which corresponds to 31.4% of the population (1). Moreover, 96.8 million sick days due to back pain were calculated in 2022, indicating high expenses for society. The Federal Statistical Office reports medical costs for diseases of the back (International Statistical Classification Codes [ICD] diagnoses M45-M54) amounting to 11.6 billion euros for 2020 (1, 2), which corresponds to 2.8% of the total healthcare costs of 431.8 billion euros.

According to the German national guideline on non-specific low back pain, in addition to somatic factors, psychological (e.g., problem-solving skills, self-efficacy expectations) and social factors (e.g., social networks, workplace) are relevant to the development and duration of the disease and should be taken into account in diagnosis and treatment (3–5). To address the complex causes of back pain, specialized multimodal treatment approaches have been developed and are now considered the gold standard (3). The national guideline also notes that interdisciplinary multimodal pain therapy (IMPT) elements should be tailored to the patient’s individual condition. Moreover, an interdisciplinary team (comprising physicians, psychologists, physiotherapists, and other professional groups) defines the goals to be achieved together with the patient at the beginning of the treatment. In addition to individual therapy sessions, the guideline suggests that the majority of therapy take place in groups in order to benefit from group dynamics (3).

Although the efficacy of IMPT has been examined in various studies (6–10), it is equally important to examine the sustainability of the treatment effects. The “IMPT with relapse prophylaxis (MMS-RFP)” study began in 2019 with the goal of evaluating the efficacy of an IMPT with additional relapse prophylaxis (RP) (11). The intervention consisted of an innovative 12-month follow-up RP to stabilise therapy elements after IMPT and examine the sustainability of treatment effects.

The study aimed to evaluate whether IMPT with RP is more effective compared with IMPT without RP. Therefore, the intervention effect on patient-relevant outcomes was examined.

The following hypotheses were examined:

- **H_1_:** The intervention reduced the days of incapacity to work
- **H_2_:** The intervention increased the functional capacity and health- related quality of life (HRQoL)

## METHODS

### Study design

**Fig. 1:**
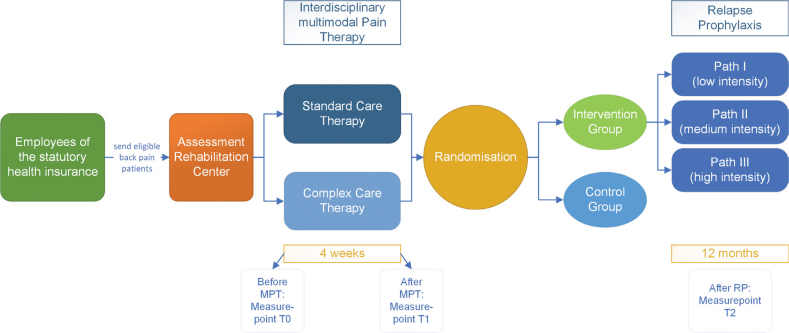
Study design.

The study was a randomised controlled trial (RCT) carried out between 2019 and 2023 in the Rehabilitation Centre “Bad Zwischenahn” (located in a North German rural area) on an outpatient basis. The participants were randomly allocated by the evaluator in a 1:1 ratio to either the control group (CG) or the intervention group (IG). The IG and the CG performed 4 weeks of IMPT in the Rehabilitation Centre, while the IG additionally received 12 months of RP (Fig. 1).

### Participants and material

The patients recruited for this study were insured by the regional health insurance provider Allgemeine Ortskrankenkasse (AOK) Lower Saxony. Only adults who were gainfully employed and entitled to sick pay were included in the study. Further inclusion criteria were a diagnosis of back pain (ICD: M40-M54; excluding M45, M46, and M49) and an incapacity to work for at least 21 days during the last 6 months before the start of the IMPT. Age over 62 and diagnoses indicating mental and behavioural disorders (ICD: F00-F99; 6 months before), malignant neoplasms (ICD: C00-C97; 12 months before incapacity to work) and injuries (ICD: S00-S99; 6 months before incapacity to work) were defined as exclusion criteria. Moreover, patients diagnosed with chronic back pain (incapacity to work for cumulatively longer than 12 weeks within the last 6 months) were excluded from the study sample, as the goal of this study was to reach patients at an early stage of their symptoms to prevent chronic pain. AOK case managers of the sick payments department contacted eligible patients for the study and sent them for an interprofessional assessment at the Rehabilitation Centre, carried out by medical and therapeutic professionals. The IMPT was divided into a standard and a complex group. Participants were allocated to the standard or complex group by an interdisciplinary team of orthopaedists, physiotherapists, and psychologists based on medical and non-medical criteria. The medical criteria included the severity of the symptoms, the work-related physical strain’ and the participants’ self-efficacy. Non-medical factors such as distance to work and socio-occupational aspects also contributed to the assignment.

Data sources used for the study were survey and claims data. Relevant claims data included days of incapacity to work to assess the primary endpoint, but also region, pharmaceuticals, therapeutic products, and remedies, as well as outpatient and inpatient care. Survey data were collected using standardised paper-based questionnaires that assessed the secondary outcomes at 3 measurement points together with sociodemographic and socioeconomic parameters. The status quo was defined through a survey conducted prior to the intervention (T0), which provided baseline values for the study. Further surveys were completed after the 4-week IMPT (T1) and again at the end of the intervention, after the 12-month RP (T2). Questionnaires at T0 and T1 were completed at the Rehabilitation Centre, but could also be sent to the evaluator if required. At T2, participants sent their completed questionnaires to the evaluator via pre-paid return envelope.

### Interventions

The IMPT, which was received by both IG and CG, consisted of a standard group and a complex group. Standard group participants received treatment 4 days a week, while complex participants were treated each working day, i.e., 5 days a week. The duration of treatment was 3–4 and 5–7 h respectively. In addition to being longer, the treatment received by the complex group focused more strongly on psychological and medical treatment. Table SI shows the IMPT elements. Modules offered for the IMPT included medical training therapy, medical or psychological consultations, psychological pain therapy seminars, progressive muscle relaxation, social medicine seminars (e.g., information concerning sick pay), back exercise, sports theory, outdoor fitness, and basic information regarding muscles and the spine, along with information on the IMPT concept.

For the RP, 3 intensity levels were offered depending on the patient’s needs:

- *Low intensity:* 1 telephone consultation and 2 booster sessions.
- *Medium intensity:* 2 telephone consultations and 3 booster sessions.
- *High intensity:* 2 phone consultations, 4 booster sessions, and monthly medical training therapy.

The RP was offered in order to stabilise the therapy elements after IMPT and intended as a continuation of treatment to reduce the likelihood of relapse. Participants were taught strategies to manage their back condition effectively, reduce the risk of pain exacerbation, and promote long-term functional improvement. Sewöster (12) described medical rehabilitation as an important part of the German healthcare system and Weier et al. (13) further explained the importance of rehabilitation aftercare for back pain patients to be better able to implement the planned behaviour in their everyday life. The RP did not include vocational rehabilitation, but offered support modules on workplace adjustment strategies and self-management techniques as part of the IMPT to support a return to work. More detailed information concerning the treatment programmes of both IMPT and RP can be found in the study protocol (11).

### Outcomes

The primary outcome investigated was days of incapacity to work. The aim was to examine whether there was a greater decrease in days incapable of work for the IG compared with the CG within 1 year of the IMPT. Secondary outcomes were functional capacity and HRQoL (health-related quality of life). It was assumed that functional capacity and HRQoL in the IG would be significantly higher and days of incapacity to work significantly lower than in the CG. Functional capacity (Hannover Functional Ability Questionnaire; HFAQ) (14–16) and HRQoL (EQ-5D-5L and EQ VAS) (17) were obtained via paper-based questionnaires. The HFAQ measures functional ability based on 12 everyday activities (e.g., Can you sit on a hard chair for one hour?). A percentage value between 0 and 100% is calculated based on the sum of 3 possible response options “no, or only with help”, “yes, but with some effort”, and “yes”. To interpret HFAQ values in more detail, comparative data from various studies can be used (14). A score between 80% and 100% corresponds to normal functional capacity and 70% indicates moderate functionality. Meanwhile, a score under 60% corresponds to the worst impairment level, describing a relevant functional impairment. The EQ-5D-5L is a generic measure of health-related quality of life (17). The health state is measured in 5 dimensions (mobility, self-care, usual activities, pain/discomfort, anxiety/depression), each with 5 response options (no problems, mild problems, moderate problems, severe problems, extreme problems/incapacity). The values of the health state index range from less than 0 to 1, with higher values indicating better health. In addition, the questionnaire includes a visual analogue scale (VAS), which is used to rate the perceived health on a scale from 0 (worst imaginable state of health) to 100 (best imaginable state of health).

### Sample size

The sample size was calculated for the primary endpoint sick days. For the included patient group with AOK insurance, an average number of 37 missed working days (SD: 58) per year was used, with the assumption that this number would decrease by 40% in the IG compared with the CG. The calculation was based on an effect size of *d* = 0.3409275. A sample size of 147 subjects per arm of the trial was required to test with a power of 80% (1−ß = 0.8) and a significance level of 5% (ɑ-error = 0.05), taking into account an intraclass correlation coefficient of 0.01 and a design effect of 1.1 with an average IMPT group size of 15.3 patients per month (18–21). Considering a 10% loss to follow-up, a total of 322 patients was needed. The sample size calculation was carried out using G*Power software (https://www.psychologie.hhu.de/arbeitsgruppen/allgemeine-psychologie-und-arbeitspsychologie/gpower).

### Randomisation and blinding

The study participants were randomly assigned to IG or CG. After the eligible participants were enrolled in the trial, the group allocation to IG or CG of each IMPT course was randomised by the evaluator. A concealed drawing procedure was used for this. The Rehabilitation Centre staff were blinded and received the information concerning the randomisation from the evaluator at the latest possible date (at the end of the third IMPT week) to reduce any influence on the participants. Accordingly, the participants were also informed as late as possible whether they would receive follow-up care. For the IMPT, standard and complex groups were formed, each with a maximum of 8 patients in parallel.

### Statistical methods

A unique identification number was assigned to all patients to pseudonymise their data and enable linking of survey and claims data. Prior to quantitative analysis, a plausibility check was conducted. Depending on the type of data (categorical or continuous), χ^2^ tests and two-sample *t*-tests for each hypothesis were carried out to test for group differences at T0, T1, and T2. In addition, differences between IG and CG with regard to change scores of primary (after IMPT–before IMPT) and secondary endpoints (T1–T0, T2–T1, T2–T0) were analysed. Subsequently, analyses of variance (ANOVA) were performed to estimate the effect of the patient allocation to the 2 IMPT and 3 RP intensities on the primary outcome sick days. Linear regression analyses were carried out to test the influence of other predictors such as age, gender, and number of sick days pre-intervention on the days of incapacity to work after IMPT. A significance level of α = 0.05 was defined.

All patients were included in the analysis, regardless of whether they discontinued RP participation (intention-to-treat analysis). The participants were analysed based on their belonging to IG or CG. A two-sided *t*-test with a significance level of 0.05 was carried out to test the primary hypothesis H_1_, “The intervention reduced the number of days of incapacity to work”. Two-sided *t*-tests with a significance level of 0.05 were also employed to examine hypothesis H_2_, “The intervention increased functional capacity and HRQoL”. Data analyses were carried out using the IBM software SPSS version 29.0.1.0 (IBM Corp, Armonk, NY, USA).

## RESULTS

### Sample characteristics

The recruitment period was 2 years, resulting in 24 IMPT courses with up to 16 participants each. The follow-up period was 12 months. The first course started in September 2019 and the last in August 2021. The last follow-up period ended in August 2022.

**Fig. 2:**
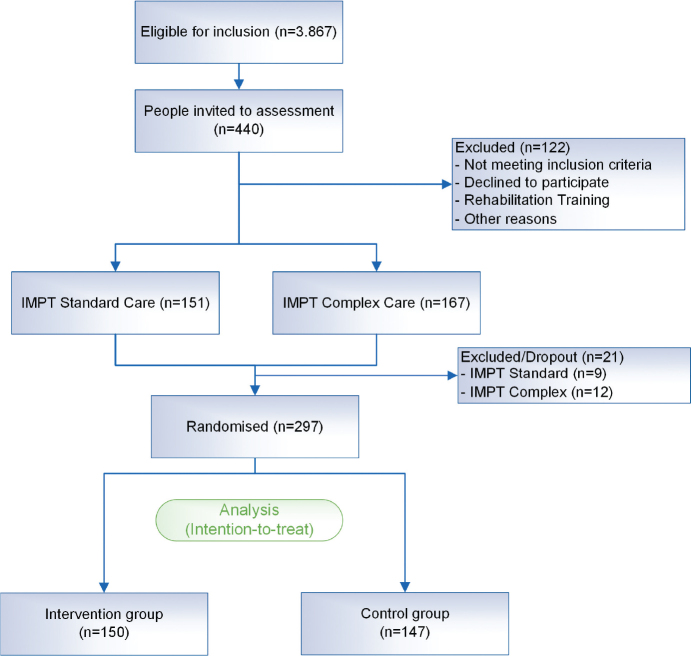
CONSORT flow diagram. IMPT: Interdisciplinary Multimodal Pain Therapy; CONSORT: Consolidated Standards of Reporting Trials.

An initial assessment was conducted with 440 participants. A total of 318 started IMPT and 21 patients discontinued the study before randomisation was carried out (Fig. 2). At 6.6%, the dropout rate was below the expected 10%. Ultimately, 297 participants were included in the intention-to-treat analysis; 150 patients were randomised to the IG and 147 to the CG. Table I presents the sample characteristics of the study population. The sample included a higher percentage of men. The average age in the IG and CG was 44 (SD: 10.99) and 46 years (SD: 9.81) respectively. Most patients were married or in a long-term relationship and lived in a rural area. Regarding education and occupation, most stated a medium educational level and vocational qualification. The monthly net household income in both groups was between €2,000 and €3,000. The χ^2^ test of the group differences showed no significant group differences for any of the sociodemographic characteristics examined, except for educational level, as more participants in the IG had a higher educational level. Table II indicates the percentage of ICD dorsopathy diagnoses (M40–M54) and more detailed subcategories separately for IG and CG. In both groups, the majority of participants were diagnosed with “Other dorsopathies” (M50–54).

**Table I:** Sample characteristics

Item	IG IMPT + RP *n* (%)	CG IMPT *n* (%)	*p*-value
Sex			
Female	54 (36.2%)	51 (35.2%)	0.85
Male	95 (63.8%)	94 (64.8%)	
Missing	1	2	
Age, mean (SD)	44.23 (10.99)	46.63 (9.81)	0.10
Missing	2	3	
Years of school education			
≤ 9	51 (34.2%)	59 (41.0%)	0.02*
10	68 (45.6%)	65 (45.1%)	
≥ 11	29 (19.5%)	17 (11.8%)	
Other degree	1 (0.7%)	3 (2.1%)	
Missing	1	3	
Vocational qualification			
None	9 (6.1%)	8 (5.6%)	0.62
Vocational training	3 (2.1%)	0 (0%)	
Completed vocational training	107 (72.3%)	99 (69.7%)	
Higher education^a^	21 (14.2%)	22 (15.5%)	
Other	2 (1.4%)	5 (3.5%)	
Declined to answer	6 (4.1%)	8 (5.6%)	
Missing	2	5	
Labour force status			
Unemployed	1 (1.0%)	0 (0%)	0.10
Worker	62 (59.0%)	61 (53.5%)	
Employee	38 (36.2%)	46 (40.4%)	
Civil servant	0 (0%)	2 (1.8%)	
Self-employed	4 (3.8%)	1 (0.9%)	
Other	0 (0%)	4 (3.5%)	
Missing	45	33	
Nationality			
German	138 (92.6%)	138 (95.2%)	0.48
Other	10 (6.7%)	7 (4.8%)	
Declined to answer	1 (0.7%)	0 (0.0%)	
Missing	1	2	

IG: intervention group; CG: control group; SD: standard deviation; IMPT: Interdisciplinary Multimodal Pain Therapy; RP: relapse prophylaxis;

* *p* < 0.05, **p < 0.01;

a degree from universities, higher education establishments, and technical colleges.

**Table II:** Included diagnoses of back pain and corresponding ICD-10 codes

ICD-Code	Main diagnoses	IG	CG
		*n* = 150	*n* = 147
M40–M54	Dorsopathies		
M40–M43	Deforming dorsopathies	26.0%	27.9%
M47–M48	Spondylopathies	0.7%	0.7%
M50–M54	Other dorsopathies	72.7%	71.4%

IG: intervention group; CG: control group; ICD: International Statistical Classification of Diseases and Related Health Problems.

### Main outcome

*Hypothesis H1.* For the period before the IMPT, no significant group difference was found between IG and CG (*p* = 0.640). Both groups had a similar number of sick days at baseline, with a mean value of 60.07 in the CG and 61.64 in the IG. Over the 12 months following the IMPT, patients had a mean (M) of 70.07 days (SD 111.50) of incapacity to work in the CG and a lower mean of 56.41 days (SD 96.24) in the IG. The *t*-test revealed no significant difference between the 2 groups (*p* = 0.259) (Table III).

**Table III:** Two-sided *t*-test analyses for days of incapacity to work

Item	IG (*n* = 150)					CG (*n* = 147)					*p*-value	SES (95% CI)
	M	MD	SD	Min	Max	M	MD	SD	Min	Max		
12 months before IMPT	61.64	55.00	28.97	6	153	60.07	56.00	28.68	0	153	0.640	0.05 (–0.28 – 0.17)
12 months after IMPT	56.41	16.00	96.24	0	365	70.07	10.00	111.50	0	365	0.259	0.13 (–0.10 – 0.36)

IG: intervention group; CG: control group; SES: standardised effect size Cohen’s *d*; 95% CI: 95% confidence interval; M: mean; SD: standard deviation; MD: median; Min: minimum; Max: maximum; IMPT: Interdisciplinary Multimodal Pain Therapy.

*Hypothesis H2.* The *t*-tests of the secondary outcomes confirmed a significant (*p* = 0.047) group difference in functional capacity between IG and CG after the intervention (at T2). The group comparison of the index score and EQ VAS (current health status) showed no significant difference (EQ-Index: *p* = 0.293; EQ VAS: *p* = 0.065) at T2. Additional results are displayed in Table IV.

**Table IV:** Two-sided *t*-tests analyses for secondary outcomes functional capacity and health-related quality of life

Item		IG				CG				*p*-value	SES (95% CI)
		*n*	M	MD	SD	*n*	M	MD	SD		
HFAQ functional capacity											
HFAQ_T0	Baseline	139	65.02	66.67	17.22	141	68.23	70.83	19.22	0.142	0.18 (–0.06 – 0.41)
HFAQ_T1	4 weeks	135	81.91	87.50	16.93	130	78.97	83.33	20.97	0.210	0.16 (–0.40 – 0.09)
HFAQ_T2	12 months	111	85.81	91.67	19.58	103	80.50	87.50	19.29	0.047*	0.27 (–0.54 – 0.00)
Health-related quality of life											
EQ5D Index_T0	Baseline	147	0.72	0.79	0.19	137	0.74	0.80	0.18	0.607	0.06 (–0.17 – 0.29)
EQ5D Index_T1	4 weeks	140	0.85	0.89	0.13	134	0.82	0.88	0.17	0.094	0.21 (–0.44 – 0.03)
EQ5D Index_T2	12 months	113	0.85	0.94	0.21	103	0.82	0.88	0.19	0.293	0.41 (–0.41 – 0.12)
VA scale											
VA-Scale_T0	Baseline	149	57.50	60	15.90	143	57.61	60	16.28	0.956	0.01 (–0.22 – 0.24)
VA-Scale_T1	4 Weeks	146	73.94	80	17.61	140	70.46	75	20.47	0.125	0.18 (–0.42 – 0.05)
VA-Scale_T2	12 Months	117	75.79	80	19.04	103	71.16	75	17.80	0.065	0.25 (–0.52 – 0.02)

IG: intervention group; CG: control group; SES: standardised effect size Cohen’s *d*; 95% CI: 95% confidence interval; HFAQ: Hannover Functional Ability Questionnaire (value range 0–100); HRQoL: health-related quality of life (EQ-5D-5L); VA scale: visual analogue scale (EQ VAS); M: mean; SD: standard deviation; MD: median;

* *p* < 0.05.

### Mean change

Table V shows the within- and between-group mean changes in the primary and secondary variables at different measurement points. The between-group difference was tested by two-sided *t*-tests. Cohen’s *d* was selected to estimate the effect size.

**Table V:** Mean change in sick days, functional capacity, and health-related quality of life

Item	IG			CG			*p*-value	SES (95% CI)
	*n*	Mean change	SD	*n*	Mean change	SD		
Sick days								
After IMPT–Before IMPT	150	–5.23	95.87	147	10.00	111.16	0.207	00.15 (–00.08 to 00.38)
HFAQ functional capacity								
T1–T0	128	17.38	18.91	124	11.19	20.59	0.013*	–0.31 (–0.56 to –0.07)
T2–T1	101	02.35	17.57	93	01.16	20.87	0.668	–0.06 (–0.34 to 0.22)
T2–T0	104	18.91	20.18	99	11.83	17.57	0.008**	–0.37 (–0.65 to –0.10)
Health-related quality of life (Index)								
T1–T0	138	0.13	0.17	125	0.08	0.18	0.039*	–0.26 (–0.50 to –0.01)
T2–T1	107	0.00	0.19	98	0.01	0.16	0.688	–0.06 (–0.33 to 0.22)
T2–T0	110	0.12	0.22	101	0.07	0.20	0.069	–0.25 (–0.52 to 0.02)
VA scale								
T1–T0	145	16.36	17.28	136	12.54	22.22	0.111	–0.19 (–0.43 to 0.04)
T2–T1	114	01.25	20.64	102	– 0.57	17.60	0.490	–0.09 (–0.36 to 0.17)
T2–T0	116	18.60	22.45	102	13.90	18.57	0.096	–0.23 (–0.49 to 0.04)

IG: intervention group; CG: control group; SES: standardised effect size Cohen’s *d*; HFAQ: Hannover Functional Ability Questionnaire (value range 0–100); HRQoL: health-related quality of life: (EQ-5D-5L); VA scale: visual analogue scale (EQ VAS); SD: standard deviation; MD: median; CI: confidence interval; IMPT: Interdisciplinary Multimodal Pain Therapy;

* *p* < 0.05,

** *p* < 0.01.

For the primary outcome, the results showed a negative mean change for the IG and a higher positive mean change for the CG after IMPT, descriptively indicating a reduction of sick leave for the IG post intervention. A significant group difference could not be identified (*p* = 0.207).

Overall, the IG displayed a higher mean change between T1 and T2 for all secondary endpoints with the exception of HRQoL. Of the secondary endpoints functional capacity and HRQoL, only functional capacity showed a significant difference between IG and CG in mean change from T0 to T1 (*p* = 0.013) and T0 to T2 (*p* = 0.008) with the highest between-group differences. In both cases, the effect size can be considered moderate according to Cohen (22).

### Subgroup analyses

For further subgroup analyses, the days of incapacity for work were used as the dependent variable. Assignment to standard and complex IMPT, RP intensity, days off work before IMPT, functional capacity at baseline, gender, and age were analysed as predictors.

**Fig. 3:**
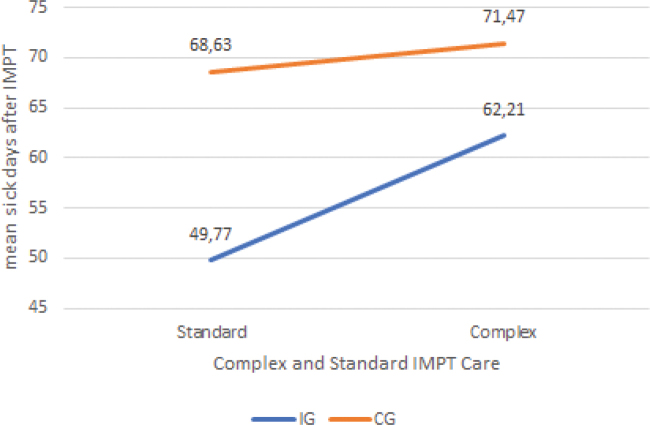
Sick days after IMPT in comparison between IG and CG in complex and standard Interdisciplinary Multimodal Pain Therapy (IMPT) care. IG: intervention group; CG: control group.

A two-factor ANOVA (factor 1: Group IG vs CG; factor 2: IMPT Standard Care vs Complex Care) found no significant interaction between group assignment and days of incapacity to work, F (1,293) = 0.16, *p* = 0.692. This indicates that there was no significant group difference between IG and CG concerning the number of sick days after IMPT. In both standard and complex IMPT, however, the sick days were higher in the CG (standard: M = 68,63; complex: M = 71,47) than the IG (standard: M = 49,77; complex: M = 62,21) (Fig. 3).

**Fig. 4:**
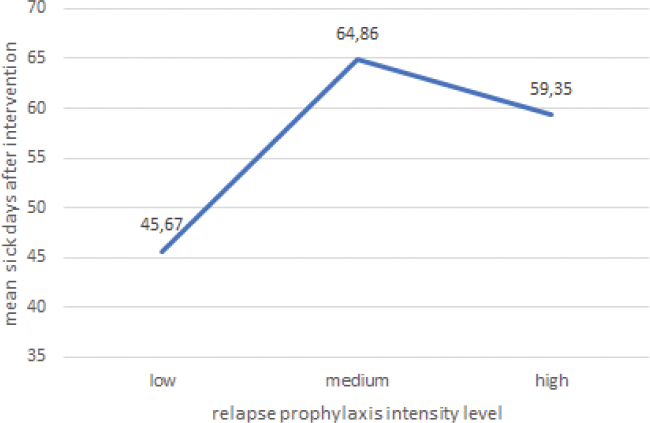
Sick days after IMPT and intensity of care in relapse prophylaxis in the IG. IMPT: Interdisciplinary Multimodal Pain Therapy. IG: intervention group.

As RP was only received by the IG, a single-factor ANOVA was conducted to estimate the influence of RP intensity (factor 1: Group allocation IG; factor 2: RP intensities low, middle, high) on days of incapacity to work. The number of sick days was lowest (M = 45,67) in path one, which provided the least follow-up care, and highest (M = 59,35) in the medium intensity RP (Fig. 4). Overall, no statistically significant difference in sick days was found between the different levels of RP intensity, F (2,147) = 0.53, *p* = 0.59. This indicates that there is no difference in return to work between the groups.

Regression analyses using sick days before IMPT, functional capacity at baseline, gender, and age as predictors showed no significant result with the binary factor group allocation, indicating no difference between IG and CG.

## DISCUSSION

### Hypotheses

Hypothesis H_1_ (the intervention reduced the days of incapacity to work) cannot be confirmed, as the groups showed no significant difference in number of sick days. However, there was a clear descriptive trend, as the IG had fewer sick days after IMPT. The *p*-value (*t*-test) is 0.26 after the IMPT, indicating a tendency towards a reduction in sick days in the IG compared with the CG. As stated before, the mean values of both groups in the pre-IMPT period are approximately the same, indicating that data is balanced across the factor group assignment and the randomisation was successful. A key methodological challenge in evaluating the first hypothesis was the wide distribution of days of incapacity to work after IMPT. Values ranged from 0 to 365 days, which is reflected in the large standard deviation. The data were not adjusted, as the outliers were real and not due to errors. Therefore, the entire sample was included in the analysis.

The second hypothesis H_2_ that the IG (M = 85.81%, SD = 19.58%) had a significantly higher functional capacity after the RP compared with the CG (M = 80.50%, SD = 19.29%) can be confirmed. According to Cohen (22), the effect is small, *d* = 0.27. Of all secondary endpoints analysed, functional capacity showed the greatest improvement in the IG compared with the CG. The functional capacity measured at T2 can be categorised as normal functional capacity (score 80–100%) in both groups and is therefore better than the moderate functional impairment (score up to 70%) measured at baseline. It should be taken into account that the HFAQ score was already higher and within the range of moderate functional capacity at the end of the IMPT (T1) and not only at the end of the RP as predicted.

Regarding patients’ HRQoL, the IG reported a higher quality of life than the CG after the intervention on both variables (EQ-5D-5L index score and EQ VAS) at T2. However, the group difference was not significant, although the *p*-value of the EQ VAS was approximately significant. It is also apparent that greater improvements in all secondary endpoints already occurred at T1 in the IG, although the effect would not have been expected until T2, as the RP phase started only after the IMPT. It seems reasonable that the participants’ expectation of receiving follow-up care had an influence here, even though the information concerning the assignment to IG or CG was given as late as possible in the last week of the IMPT (23, 24).

### Subgroup analyses

Through subgroup analyses, further factors influencing the dependent variable days of incapacity for work were investigated. First, the IG had fewer sick days both in total and in the standard and complex IMPT subgroups compared with the CG. Second, the result that the complex group had more sick days could be due to the fact that participants with more severe back pain were assigned to the IMPT with higher care (complex group). Although further regression analyses could not prove a significant result for the group factor, it became clear that sick days before IMPT and functional capacity at baseline had the strongest influence. Age and gender had no effect on sick days after IMPT.

### Strengths, limitations, and generalisability

A strength of the study was the RCT design, as it is the design of choice for examining interventions. The advantages are that the selection bias is reduced and, due to randomisation, possible confounders are assigned evenly to the study groups (25–27). In addition, special requirements apply to RCT studies, e.g., registered study protocols (25, 28). The present study was registered and the study protocol was published beforehand to ensure transparency.

The validity of the study is also supported by the use of standardised measurement instruments. However, self-report scales are a subjective measure and therefore prone to other biases. They can predict real behaviour and preferences only to a limited extent, as response biases, social desirability, and acquiescence can occur when answering the questions (29).

With regard to the data used, one of the main advantages of claims data is that missing data are avoided and full data availability is ensured. To ensure high data quality, comprehensive plausibility checks and validations were carried out. Although errors may have occurred when assigning the ICDs for the periods of incapacity for work, claims data can be regarded as the most reliable source of information, as patients often have trouble remembering the specific time of sickness-related absence from work. However, claims data are not primarily collected for research purposes (30) and corresponding limitations must therefore be considered (31). Finally, it can be positively emphasised that the required sample size was achieved to test the intervention effect of the primary endpoint with sufficient power. Particularly during the COVID-19 pandemic, recruitment difficulties had to be addressed. Fortunately, the study centre and medical staff were able to keep the study conditions constant and confounding influence during IMPT was limited. When interpreting the study results, however, it must also be considered that the COVID-19 pandemic may have had an impact on the return to work. The present sample was made up predominantly of men who completed vocational training and had the labour force status “worker”, indicating more physical work. In addition, more participants in the IG stated a higher level of education. Although most participants remained in the same job, around 12% changed employment in the 12 months following the IMPT. It was not documented in detail whether the job change involved a less physically demanding activity, and job-related physical strain in some cases could not be determined based on available information concerning participants’ occupations. Regarding the RP, the process evaluation revealed a number of difficulties in implementation of the follow-up treatment. In order to assess the actual benefit of the intervention RP, it must be taken into account that participation rates during the RP were lower than estimated. The Rehabilitation Centre staff made considerable efforts to increase participation during RP. For example, several invitations and reminders to participate in scheduled booster sessions were sent out. Nonetheless, rates remained low on average. The evaluation therefore revealed the potential to improve participation during the RP in order to improve assessment of the intervention effect of RP. Finally, determining participation in the RP and enhancing patient motivation to participate are relevant factors in order to be able to evaluate the actual benefit in subsequent studies.

### Comparisons with other studies

As previously mentioned, the effectiveness of IMPT has been assessed in various studies. Elbers et al. (6) addressed the issue that content and duration of multimodal programmes can vary and conducted a review and meta-analysis to compare the outcomes of different programmes. Based on the included studies, a significant improvement in chronic primary musculoskeletal pain could be demonstrated in the majority of the programmes. Hüppe et al. (32) found in 2019 that a two-year health programme with multimodal intervention aspects led to small positive long-term effects. In an RCT, Anema et al. (33) found that a workplace intervention, as part of multidisciplinary rehabilitation, resulted in participants returning to work in less time than the non-intervention group workers. In a Cochrane systematic review and meta-analysis, Kamper et al. (7) concluded that multidisciplinary biopsychosocial rehabilitation programmes were more effective in reducing both pain and disability compared with the usual care and physical treatments. For work-related outcomes, however, multidisciplinary rehabilitation appeared to be more effective than physical treatment, but demonstrated no improvement over usual care.

More recent studies have not been able to prove the superiority of IMPT over standard treatment. Langagergaard et al. (34) and Pedersen et al. (35) reported the results of an RCT study comparing the return to work rate between employees who received a brief or multidisciplinary intervention, with the multidisciplinary intervention failing to achieve better return to work rates. Another RCT conducted by Fisker et al. (36) in 2022 to investigate incapacity for work in relation to the effectiveness of a 12-week multidisciplinary vocational rehabilitation programme in Denmark showed no superiority of the intervention over standard care. However, it should be taken into account that both groups received treatment at the centre and the usual care in similarly conducted studies may be less extensive. There are only a few studies investigating RP in the context of multimodal back pain treatments. Schmidt et al. (37) found, in a single-centre trial, that an integrated rehabilitation programme combining 3 weeks of inpatient stay with 12 weeks of home-based activities and additional inpatient booster sessions was no more effective at the 26-week follow-up than an established 4-week inpatient rehabilitation programme. The 1-year follow-up study (38) confirmed this result. In contrast, Deck et al. (39) found significant improvements for the IG in terms of participation (disease-related impairments in everyday life) and functional impairment (assessed by HFAQ) 12 months after the rehabilitation programme. Schramm et al. (40) continued the investigation of an intensive follow-up strategy for rehabilitants with chronic back pain with a further 24-month catamnesis. The study was not able to prove that the significant group differences were maintained over 20 years. Methodological challenges with regard to aftercare concepts were discussed as one of the possible reasons. The authors also noted that the long-term implementation of a healthy lifestyle requires stable social and intrapersonal resources and, if necessary, individual case management in order to achieve a long-term sustainable effect through the intervention. The latter could improve the low patient motivation during the RP phase that we also observed. Elbers et al. (6) included the studies by Tavafian et al. (41) and Monticone et al. (42) as cohorts with extensive follow-up modules. Even though there were differences in the design of the follow-up care and IMPT (e.g., only the IG receiving IMPT) both approaches emphasised that treatment does not end with the acute intervention phase, but requires continuous support and promotion of self-management skills in order to achieve lasting positive effects.

One major challenge when comparing study results is the heterogeneous concept of interdisciplinary multimodal pain therapy and relapse prevention programmes, under which various forms of therapy are summarised. In addition, as mentioned earlier, the intensity and duration of the treatment provided varies. By further examining the results of this study, it is also noticeable that the improvements in the secondary endpoint, functional capacity, were reflected only to a limited extent in the primary endpoint sick days.

### Conclusion

Findings of this study show improvements in the secondary outcomes functional ability and HRQoL, which are based on self-reporting and show better results at the end of the study. However, the outcomes indicate that further studies are needed to gain knowledge to sustainably reduce days off work due to back pain.

## Supplementary Material



## References

[1] Ashrafian S, Schüssel K, Schlotmann A, Weirauch H, Brückner G, Schröder H, et al. Gesundheitsatlas Deutschland – Rückenschmerzen 2023. 10.4126/FRL01-006453981

[2] Statistisches Bundesamt (Destatis). Genesis online Tabelle 23631-0001 Krankheitskosten, Krankheitskosten je Einwohner: Deutschland, Jahre, Krankheitsdiagnosen. 2022 [cited 2024 Apr 15]. Available from: https://www-genesis.destatis.de/genesis//online?operation=table&code=23631-0001

[3] Bundesärztekammer (BÄK), Kassenärztliche Bundesvereinigung (KBV), Arbeitsgemeinschaft der Wissenschaftlichen Medizinischen Fachgesellschaften (AWMF). Nationale Versorgungsleitlinie Nicht-spezifischer Kreuzschmerz – Langfassung. 2. Auflage. Version 1. 2017.

[4] Bialas P. Das biopsychosoziale Krankheitsmodell. Schmerzmed 2022; 38: 56–58. 10.1007/s00940-022-3405-5

[5] Kaiser U, Treede RD, Sabatowski R. Multimodal pain therapy in chronic noncancer pain: gold standard or need for further clarification? Pain 2017; 158: 1853–1859. 10.1097/j.pain.0000000000000902 28328572

[6] Elbers S, Wittink H, Konings S, Kaiser U, Kleijnen J, Pool J, et al. Longitudinal outcome evaluations of Interdisciplinary Multimodal Pain Treatment programmes for patients with chronic primary musculoskeletal pain: a systematic review and meta-analysis. Eur J Pain 2022; 26: 310–335. 10.1002/ejp.1875 34624159 PMC9297911

[7] Kamper SJ, Apeldoorn AT, Chiarotto A, Smeets RJEM, Ostelo RWJG, Guzman J, et al. Multidisciplinary biopsychosocial rehabilitation for chronic low back pain. Cochrane Database Syst Rev 2014; 2014: CD000963. 10.1002/14651858.CD000963.pub3 25180773 PMC10945502

[8] van Middelkoop M, Rubinstein SM, Kuijpers T, Verhagen AP, Ostelo R, Koes BW, et al. A systematic review on the effectiveness of physical and rehabilitation interventions for chronic non-specific low back pain. Eur Spine J 2011; 20: 19–39. 10.1007/s00586-010-1518-3 20640863 PMC3036018

[9] Jensen IB, Busch H, Bodin L, Hagberg J, Nygren Å, Bergström G. Cost effectiveness of two rehabilitation programmes for neck and back pain patients: a seven year follow-up. Pain 2009; 142: 202–208. 10.1016/j.pain.2008.12.015 19217717

[10] Müller G, Pfinder M, Clement M, Kaiserauer A, Deis G, Waber T, et al. Therapeutic and economic effects of multimodal back exercise: a controlled multicentre study. J Rehabil Med 2019; 51: 61–70. 10.2340/16501977-2497 30406268

[11] Krueger K, Schmetsdorf J, Pavlovic M, Runde W, Zechel G, Hemken N, et al. Evaluation of a multimodal pain therapy approach with relapse prophylaxis for back pain (MMS-RFP study): a study protocol for a cluster randomised controlled trial. BMJ Open 2023; 13: e067412. 10.1136/bmjopen-2022-067412 PMC10314416 37349102

[12] Sewöster D. Viel Bewegung in der Reha-Nachsorge. B & G 2019; 35: 276–278. 10.1055/a-0985-3437

[13] Weier L, Steinhäuser J, Träder JM, Deck R. Hausarztzentrierte Rehabilitationsnachsorge bei chronischen Rückenschmerzen. Rehabilitation 2021; 60: 195–203. 10.1055/a-1286-2595 33477195

[14] Kohlmann T, Raspe H. Der Funktionsfragebogen Hannover zur alltagsnahen Diagnostik der Funktionsbeeinträchtigung durch Rückenschmerzen (FFbH-R). Rehabilitation 1996; 35: I–VIII. 8693180

[15] Thomas E, Silman AJ, Croft PR, Papageorgiou AC, Jayson MI, Macfarlane GJ. Predicting who develops chronic low back pain in primary care: a prospective study. BMJ 1999; 318: 1662–1667. 10.1136/bmj.318.7199.1662 10373170 PMC28145

[16] Croft PR, Macfarlane GJ, Papageorgiou AC, Thomas E, Silman AJ. Outcome of low back pain in general practice: a prospective study. BMJ 1998; 316: 1356–1359. 10.1136/bmj.316.7141.1356 9563990 PMC28536

[17] Graf von der Schulenburg JM, Claes C, Greiner W, Uber A. Die deutsche Version des EuroQol-Fragebogens. Z Gesundh Wiss 1998; 6: 3–20.

[18] Eldridge SM, Ashby D, Kerry S. Sample size for cluster randomized trials: effect of coefficient of variation of cluster size and analysis method. Int J Epidemiol 2006; 35: 1292–1300. 10.1093/ije/dyl129 16943232

[19] Dreyhaupt J, Mayer B, Kaluscha R, Muche R. Cluster-randomisierte Studien: Methodische und praktische Aspekte. Rehabilitation 2020; 59: 54–61. 10.1055/a-0801-5697 30674047

[20] Rutterford C, Copas A, Eldridge S. Methods for sample size determination in cluster randomized trials. Int J Epidemiol 2015; 44: 1051–1067. 10.1093/ije/dyv113 26174515 PMC4521133

[21] Lorenz E, Köpke S, Pfaff H, Blettner M. Cluster-randomized studies. Dtsch Arztebl Int 2018; 115: 163–168. https://di.aerzteblatt.de/int/archive/article/196558 29587960 10.3238/arztebl.2018.0163 PMC5881078

[22] Cohen J. Statistical power analysis for the behavioral sciences. 2nd ed. Hillsdale, NJ: Lawrence Erlbaum; 1988.

[23] Kessler K, Hüppe M, Roesner A. Erwartete Einflussbereiche der Disziplinen Medizin, Psychologie und Physiotherapie in der Schmerztherapie: Eine Umfrage unter Berufszugehörigen. Schmerz 2023; 37: 274–280. 10.1007/s00482-023-00726-5 37280448

[24] Maser D, Müller D, Bingel U, Müßgens D. Ergebnisse einer Pilotstudie zur Rolle der Therapieerwartung bei der interdisziplinären multimodalen Schmerztherapie bei chronischem Rückenschmerz. Schmerz 2022; 36: 172–181. 10.1007/s00482-021-00590-1 34618234 PMC9156493

[25] Saldanha IJ, Skelly AC, Ley KV, Wang Z, Berliner E, Bass EB, et al. Inclusion of nonrandomized studies of interventions in systematic reviews of intervention effectiveness: an update. Rockville, MD: Agency for Healthcare Research and Quality; 2022. 10.23970/AHRQEPCMETHODSGUIDENRSI 36153934

[26] Deeks JJ, Dinnes J, D’Amico R, Sowden AJ, Sakarovitch C, Song F, et al. Evaluating non-randomised intervention studies. Health Technol Assess 2003; 7: iii–x, 1–173. 10.3310/hta7270 14499048

[27] Hernán MA, Hernández-Díaz S, Robins JM. A structural approach to selection bias. Epidemiology 2004; 15: 615–625. 10.1097/01.ede.0000135174.63482.43 15308962

[28] Sumbauer H. Kriterien zur Qualitätsbewertung von randomisierten kontrollierten Studien (RCTs). DO 2020; 18: 4–12. 10.1055/a-0966-5252

[29] Moosbrugger H, Brandt H. Itemkonstruktion und Antwortverhalten. In: Moosbrugger H, Kelava A, editors. Testtheorie und Fragebogenkonstruktion. 3., vollständig neu bearbeitete, erweiterte und aktualisierte Auflage. Berlin: Springer; 2020: p. 67–89. 10.1007/978-3-662-61532-4_4

[30] Graf von der Schulenburg JM, Lange A, Neubauer S, Zeidler J. Prozessorientierter Leitfaden für die Analyse und Nutzung von Routinedaten der Gesetzlichen Krankenversicherung. Nomos eLibrary; 2017. 10.5771/9783845281193

[31] Swart E, Ihle P, Gothe H, Matusiewicz D, editors. Routinedaten im Gesundheitswesen: Handbuch Sekundärdatenanalyse: Grundlagen, Methoden und Perspektiven. 2., vollständig überarbeitete und erweiterte Auflage. Bern, München: Verlag Hans Huber; ciando; 2014.

[32] Hüppe A, Zeuner C, Karstens S, Hochheim M, Wunderlich M, Raspe H. Feasibility and long-term efficacy of a proactive health program in the treatment of chronic back pain: a randomized controlled trial. BMC Health Serv Res 2019; 19: 714. 10.1186/s12913-019-4561-8 31639016 PMC6805578

[33] Anema JR, Steenstra IA, Bongers PM, Vet HCW de, Knol DL, Loisel P, et al. Multidisciplinary rehabilitation for subacute low back pain: graded activity or workplace intervention or both? A randomized controlled trial. Spine 2007; 32: 291–298; discussion 299–300. 10.1097/01.brs.0000253604.90039.ad 17268258

[34] Langagergaard V, Jensen OK, Nielsen CV, Jensen C, Labriola M, Sørensen VN, et al. The comparative effects of brief or multidisciplinary intervention on return to work at 1 year in employees on sick leave due to low back pain: a randomized controlled trial. Clin Rehabil 2021; 35: 1290–1304. 10.1177/02692155211005387 33843296

[35] Pedersen KKW, Langagergaard V, Jensen OK, Nielsen CV, Sørensen VN, Pedersen P. Two-year follow-up on return to work in a randomised controlled trial comparing brief and multidisciplinary intervention in employees on sick leave due to low back pain. J Occup Rehabil 2022; 32: 697–704. 10.1007/s10926-022-10030-1 35147899

[36] Fisker A, Langberg H, Petersen T, Mortensen OS. Effects of an early multidisciplinary intervention on sickness absence in patients with persistent low back pain: a randomized controlled trial. BMC Musculoskelet Disord 2022; 23: 854. 10.1186/s12891-022-05807-7 36088313 PMC9463744

[37] Schmidt AM, Schiøttz-Christensen B, Foster NE, Laurberg TB, Maribo T. The effect of an integrated multidisciplinary rehabilitation programme alternating inpatient interventions with home-based activities for patients with chronic low back pain: a randomized controlled trial. Clin Rehabil 2020; 34: 382–393. 10.1177/0269215519897968 31912752 PMC7029437

[38] Schmidt AM, Laurberg TB, Moll LT, Schiøttz-Christensen B, Maribo T. The effect of an integrated multidisciplinary rehabilitation programme for patients with chronic low back pain: long-term follow up of a randomised controlled trial. Clin Rehabil 2021; 35: 232–241. 10.1177/0269215520963856 33040598 PMC7874370

[39] Deck R, Schramm S, Hüppe A. Begleitete Eigeninitiative nach der Reha (“neues Credo”) – ein Erfolgsmodell? Rehabilitation 2012; 51: 316–325. 10.1055/s-0031-1291279 22473476

[40] Schramm S, Hüppe A, Jürgensen M, Deck R. Begleitete Eigeninitiative nach der Reha (“Neues Credo”) – Langzeitergebnisse der quasiexperimentellen Interventionsstudie. Rehabilitation 2014; 53: 297–304. 10.1055/s-0033-1358388 24399285

[41] Tavafian SS, Jamshidi AR, Mohammad K. Treatment of chronic low back pain: a randomized clinical trial comparing multidisciplinary group-based rehabilitation program and oral drug treatment with oral drug treatment alone. Clin J Pain 2011; 27: 811–818. 10.1097/AJP.0b013e31821e7930 21642845

[42] Monticone M, Ambrosini E, Rocca B, Cazzaniga D, Liquori V, Foti C. Group-based task-oriented exercises aimed at managing kinesiophobia improved disability in chronic low back pain. Eur J Pain 2016; 20: 541–551. 10.1002/ejp.756 26198386

