# Supplementary material for: EVALUATING RELAPSE PROPHYLAXIS IN ADDITION TO INTERDISCIPLINARY MULTIMODAL PAIN THERAPY FOR BACK PAIN: A RANDOMISED CONTROLLED TRIAL
Source: J Rehabil Med. 2025 Aug 20;57:42088. doi: 10.2340/jrm.v57.42088 (PMC12379723; doi:10.2340/jrm.v57.42088)
Supplement: Supplementary file 1 [file JRM-57-42088-s1.pdf]

Supplementary material has been published as submitted. It has not been copyedited, or typeset by Jpurnal of Rehabilitation Medicine

Table SI. Interdisciplinary Multimodal Pain Therapy Treatment Schedule

IPC: Individual psychological consultation; PMR: progressive muscle relaxation; SML: social medicine lectures; MD: medical doctor; P: psychologist.

**Time**	**Monday**		**Tuesday**		**Wednesday**		**Thursday**		**Friday**	
	Group A	Group B	Group A	Group B	Group A	Only Group B	Group A	Group B	Group A	Group B
**08:00**	IPC	IPC		IPC		IPC	IPC		Team meeting	
**09:00**	Welcoming (only first day)			Back exercise		Aqua jogging	Motivation	Break		
**09:30**	Basic information	Work hardening							Back exercise	
**09:45**			Back exercise	Break			Break	Motivation		
**10:30**	PMR	Basic information	Sports theory	PMR		Work hardening	Lecture about social medicine, FAQ ‘s		SML	Work hardening
**10:45**										
**11:30**	Break		Break			Break	Break		Break	
**11:45**	Work hardening	SML	SML	Sports theory		SML	Back exercise		Work hardening	PMR
**12:45**	Break		Break			Break	Break		Break	
**13:30**	Medical training therapy	Medical training therapy				Outdoor fitness	Medical training therapy		Medical training therapy	
**14:30**										
**15:00**										
**15:30**		Consultation MD or P		Consultation MD or P		Consultation MD or P		Consultation MD or P		

Table SII. Additional sample characteristics

IG: Intervention Group; CG: Control Group; SD: standard deviation; IMPT: Interdisciplinary Multimodal Pain Therapy; RP: Relapse Prophylaxis.

	IG<br>IMPT + RP<br>n (%)	CG<br>IMPT<br>n (%)	p-Value
**Relationship status**			
Unmarried and no partner	29 (20.0%)	26 (18.7%)	
Married or partner	116 (80.0%)	113 (81.3%)	0.78
Missing	5	8	
**How many people currently reside in your household. including yourself?**			
1	19 (13.0%)	19 (13.3%)	
2	49 (33.6%)	46 (32.2%)	
3	32 (21.9%)	38 (26.6%)	0.95
4+	46 (31.5%)	40 (28.0%)	
Missing	4	4	
**How many children under 18 currently reside in your household?**			
0	77 (56.2%)	84 (64.1%)	
1	30 (21.9%)	22 (16.8%)	
2	19 (13.9%)	18 (13.7%)	0.21
3+	11 (8.0%)	7 (5.4%)	
Missing	13	16	
**Country of birth**			
Germany	119 (81.0%)	120 (83.9%)	
Other	28 (19.0%)	23 (16.1%)	0.51
Missing	3	4	
**Place of residence**			
Rural	97 (66.0%)	87 (60.8%)	
Urban	48 (32.7%)	55 (38.5%)	0.52
Declined to answer	2 (1.4%)	1 (0.7%)	
Missing	3	4	
**Home ownership status**			
Owner	74 (50.0%)	94 (66.2%)	0.005**

	IG<br>IMPT + RP<br>n (%)	CG<br>IMPT<br>n (%)	p-Value
Tenant	74 (50.0%)	48 (33.8%)	
Missing	2	5	
**Type of residence**			
Apartment	53 (35.8%)	38 (26.8%)	
House	95 (64.2%)	104 (73.2%)	0.10
Missing	2	5	
**Monthly net household income**			
Under 500 Euro	0 (0%)	1 (0.7%)	
500 - 1.000 Euro	1 (0.7%)	3 (2.1%)	
1.000 - 2.000 Euro	38 (25.8%)	42 (30.0%)	
2.000 - 3.000 Euro	59 (40.1%)	46 (32.8%)	
3.000 - 4.000 Euro	24 (16.3%)	25 (17.9%)	0.86
4.000 - 5.000 Euro	18 (12.2%)	15 (10.7%)	
5.000 - 6.000 Euro	5 (3.4%)	5 (3.5%)	
6.000 Euro or higher	2 (1.4%)	3 (2.1%)	
Missing	3	7	
